# Starting the conversation

**DOI:** 10.1038/s43856-022-00099-3

**Published:** 2022-04-07

## Abstract

Listening to the different communities that we serve, and promoting dialogue between them, are important goals for us as a journal. To further these aims, *Communications Medicine* is now publishing a new article type that will help us capture and amplify the voices of all those involved in clinical, translational and public health research, and allow them to more easily hear each other’s point of view.

When *Communications Medicine* published its first articles in June 2021, our accompanying Editorial stated that one of our aims was to encourage discussion and foster collaboration across research fields and medical specialties through the publication of commentary articles. We also pledged to promote diverse representation and to provide a platform for all participants in research and healthcare, including those who ultimately could be impacted most significantly by the research that we publish, those experiencing or living with disease.

Since our launch, we have published Comments on a range of topics, with authors from a number of different fields and communities, from clinicians working on ovarian cancer, to scientists studying malaria immunity and vaccines, or authors working in the pharmaceutical industry. We have also published a piece on long COVID drawing insight from the author’s own lived experience, research and advocacy work. We feel, however, that the Comment article format limits us to hearing one side of the story, from a single author or group of authors often working closely together. With this in mind, we are introducing a new article type called Viewpoint, in which at least two authors with different backgrounds or research interests will comment on the same topic. We hope that this article type will allow us to ensure everyone has a voice, and that this will reach communities that would not normally hear it, hopefully catalysing crosstalk and collaboration between them.

While we will seek the views of key opinion leaders in the field—whether these are scientists, clinicians or other professionals—we feel it is equally important to amplify the voices of more junior researchers. We will also endeavour to include the voices of patients or patient advocates on the issues that matter to them. Promoting diversity and inclusion will be core aims for this series of Viewpoint articles, both in terms of the authors we ask to contribute and the topics that are covered. Our priority is to capture a range of opinions and perspectives on the topic at hand, as we believe diversity of thought is key to driving scientific and medical progress.

Today, we publish our first two Viewpoint articles. One is on the recent clinical and translational advances in the field of oncolytic virotherapy, an area of oncology that has seen exciting progress lately but with a number of challenges to address before oncolytic viruses can become an established treatment modality for cancer. This captures the views of both clinicians and scientists working in this field, from the US, Canada and Germany. The second Viewpoint is on global disparities in access to cancer care and proposes steps to close these gaps in low- and middle-income countries. This includes contributions from an epidemiologist and clinicians based in Uganda, India, the UK and the US.

We hope that our series of Viewpoint articles—alongside our other commentary articles—will be interesting to our readers and will stimulate discussion and crosstalk between fields and professions. If you have an idea for a topical area to cover in one of these articles, then please feel free to get in touch. You can find our contact details on the Editors page of our website, or reach us on Twitter (@CommsMedicine).

